# Supplementary material for: Optimal coding and neuronal adaptation in economic decisions
Source: Nat Commun. 2017 Oct 31;8:1208. doi: 10.1038/s41467-017-01373-y (PMC5662730; doi:10.1038/s41467-017-01373-y)
Supplement: Supplementary file 1 — Supplementary Information [file 41467_2017_1373_MOESM1_ESM.pdf]

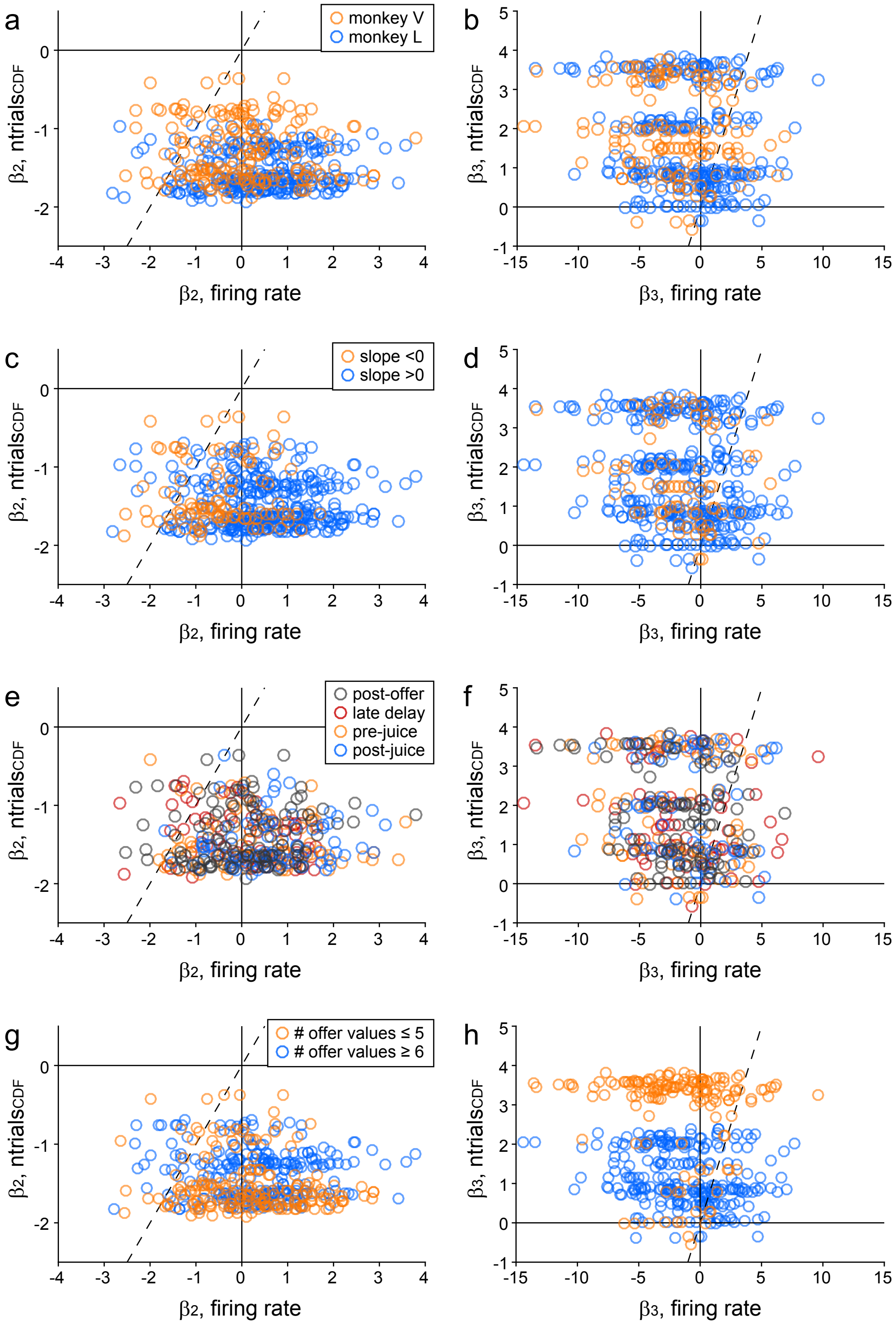

Supplementary Figure 1. Quasi-linear coding of offer values, control analyses. **ab.** Individual monkeys. Both panels illustrate the same data shown in Fig.2, and different colors indicate neuronal responses from the two monkeys. **cd.** Sign of the encoding. The neuronal encoding of value could be positive (higher firing rates for higher values, or slope>0) or negative (higher firing rates for lower values, or slope<0). In this case, different colors indicate neuronal responses with the two slope signs. **ef.** Individual time windows. In Fig.2ab, we pooled data from different time windows. Here different colors indicate responses from individual time windows. For clarity, we included in panels (ef) only responses from the four primary time windows. **gh.** Number of offer values. The number of offer values varied for different responses, and was typically lower for offer value A than for offer value B responses (see Fig.1). Here different colors indicate that the number of value offered was low (≤5) or high (≥6).

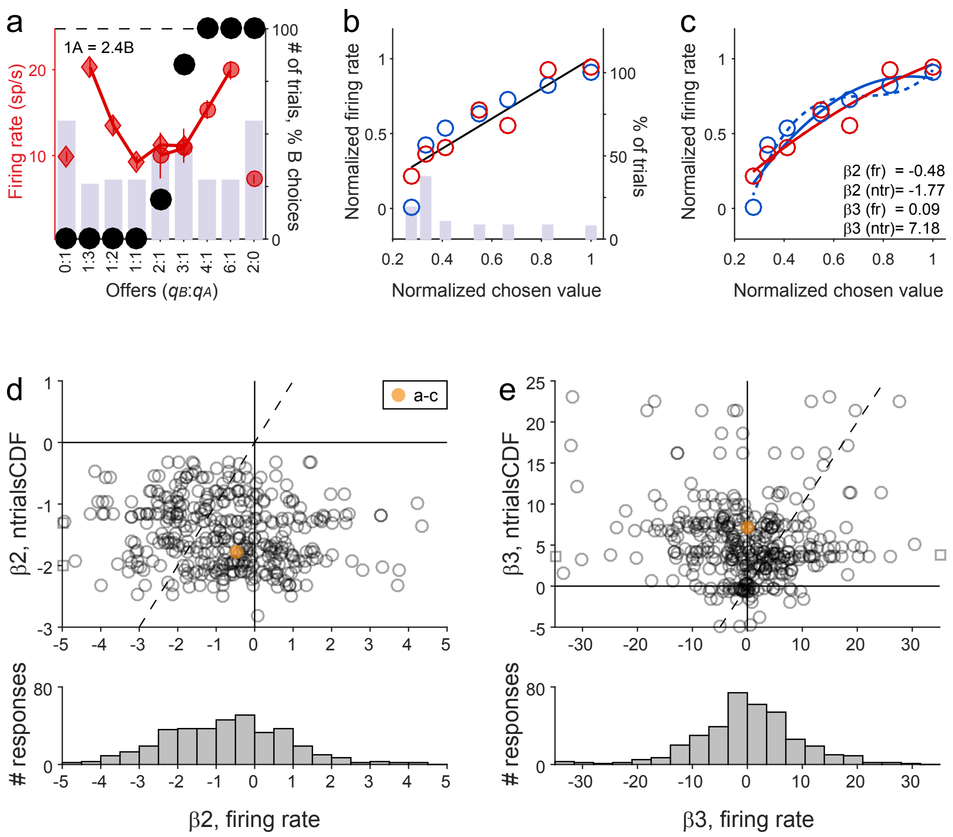

Supplementary Figure 2. Quasi-linear coding of chosen value. **a-c.** Example. Same format as in Fig.1. **de.** Population analysis (N = 370). Same format as in Fig.2.

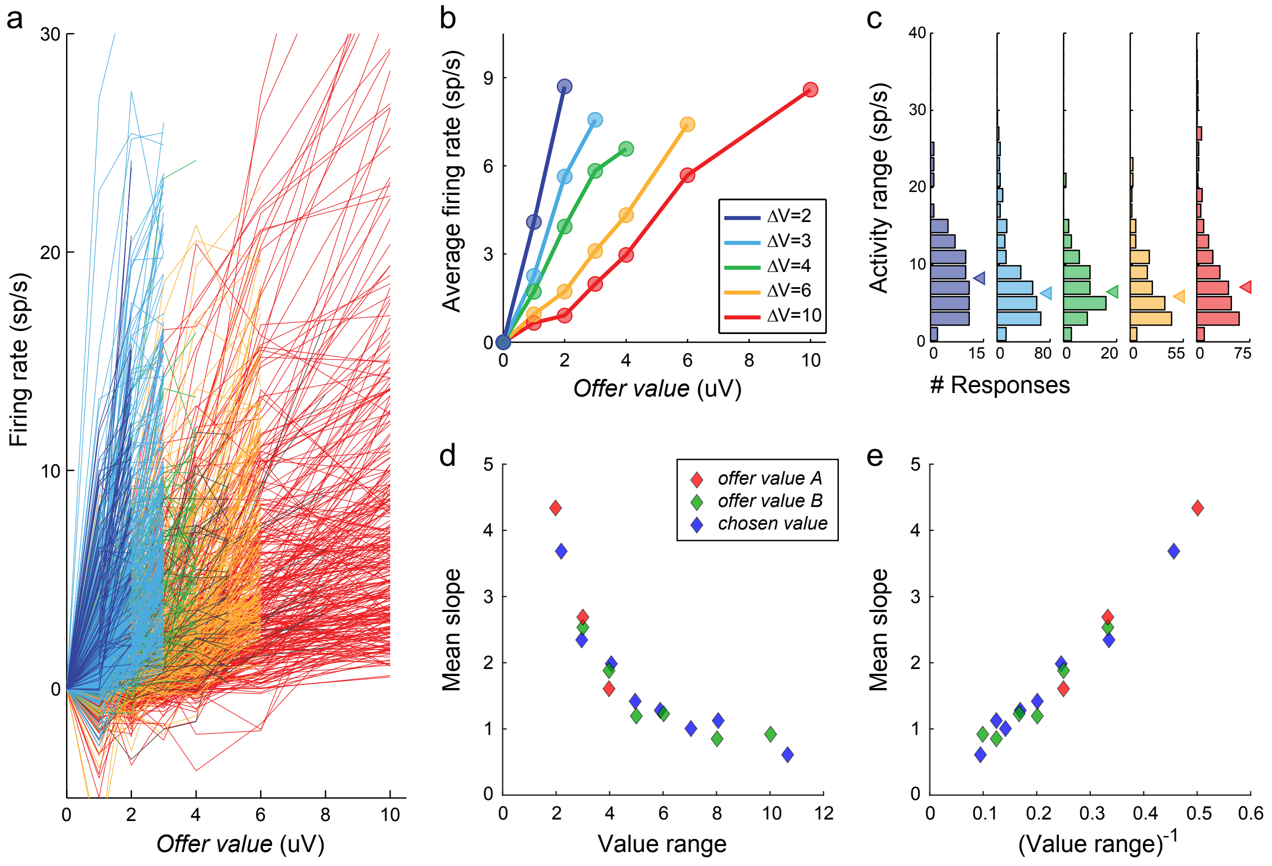

Supplementary Figure 3. Range adaptation in OFC. **ab.** Individual responses and population averages. Each line in (a) represents one neuronal response. Responses were baseline-subtracted and color-coded according to the range of values offered for the encoded juice. Responses with positive encoding (increasing firing rates for increasing values) and negative encoding (decreasing firing rate for increasing values) were pooled. Each color group presented a wide distribution of firing rates. However, range adaptation became clear once responses were averaged separately for each group (panel b). **c.** Distribution of activity ranges. Each histogram illustrates the distribution of activity ranges recorded with the different value ranges. **de.** Mean tuning slope. In (d), each symbol represents the tuning slope (y-axis) averaged across all the neuronal responses recorded with a given value range (x-axis). The three colors indicate different groups of cells (see legend). In (e), the same data points are plotted against the inverse value range (x-axis). Reproduced from [6].

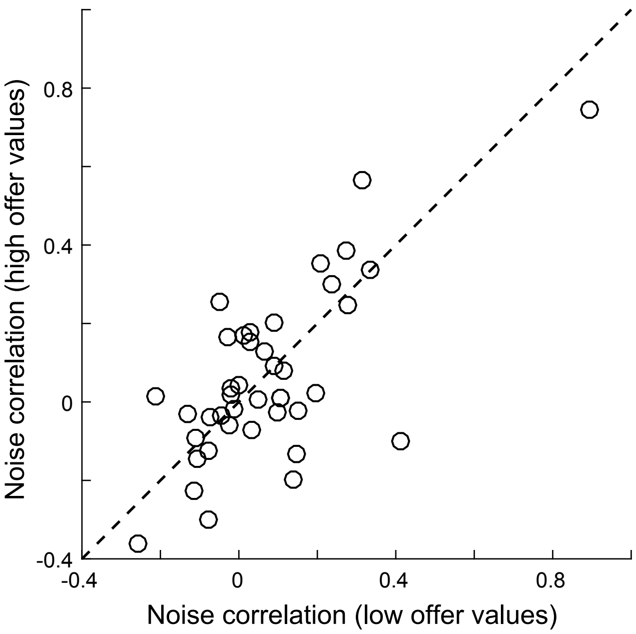

Supplementary Figure 4. The theoretical argument showing that range adaptation ensures maximum payoff assumes that noise correlations (ξ in Eq.2) do not depend on the slope of the encoding (t in Eq.2). In other words, we assumed that range adaptation would not affect noise correlations. Noise correlations in Exp.1 were analyzed in a previous study, where we found a weak but significant relation between the baseline firing rates and noise correlations (Fig.3c in [30]). In other words, pairs of neurons with higher firing rates were slightly more correlated. However, this finding is not directly relevant to the issue of interest here, namely whether noise correlation depends on the firing rates given a pair of cells. To address this issue, we re-examined the same data set focusing on pairs of *offer value* cells recorded simultaneously, associated with the same juice and with the same coding sign (N = 41 pairs; see [30] for detail). For each cell pair, we divided trials based on whether the offer value was above or below the median offer value in that session. (Trials in which the value was exactly equal to the median were excluded.) We then computed the noise correlation separately for the two groups of trials. The scatter plot illustrates the results obtained for this population. Each data point represents one cell pair, and the x- and y-axes represent the noise correlation measured in low-value trials (ξ_{low}) and in high-value trials (ξ_{high}), respectively. Notably, noise correlations did not differ systematically between the two groups of trials (median(ξ_{high} - ξ_{low}) = 0.001; p = 0.62, paired t-test). Similar results were obtained by inverting the axes for pairs of cells with negative encoding (median(ξ_{high} - ξ_{low}) = 0.006; p = 0.47, paired t-test) any by excluding pairs of cells from the same electrode (median(ξ_{high} - ξ_{low}) = 0.003; p = 0.33, paired t-test).

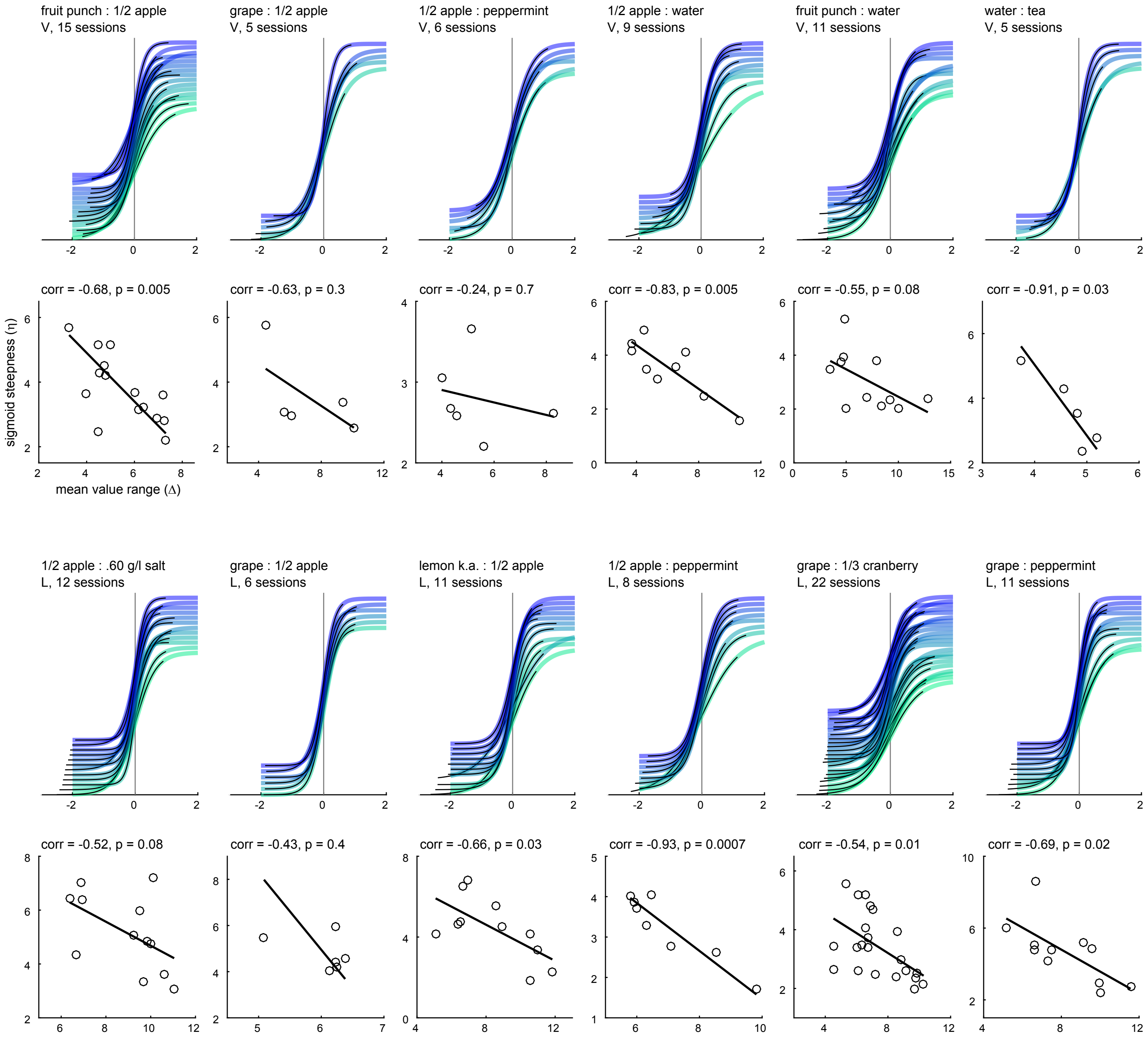

Supplementary Figure 5. Relation between sigmoid steepness (η) and value range (Δ), by juice pair. Fig.6 includes sessions with different juice pairs, with different typical values for ρ. In principle, choice variability could vary from juice pair to juice pair in a way that induces the relation between η and Δ. To address this issue, we divided sessions in different sets based on the juice pair. Considering only sets with ≥5 sessions, our data included 12 viable sets (6 from each monkey). Here we illustrate the analysis restricted to individual data sets. For each set, the top panel illustrates the fitted sigmoids (equivalent to Fig.5) and the bottom panel illustrates the relation between η and Δ (equivalent to Fig.6). For each set we indicate explicitly the juice pair, the animal (V or L) and the number of sessions available. In the bottom panel, we also indicate the correlation coefficient with its p value (as in Fig.6). Black lines illustrate the result of Deming's regressions. Notably, the negative correlation between η and Δ can be observed for each set.

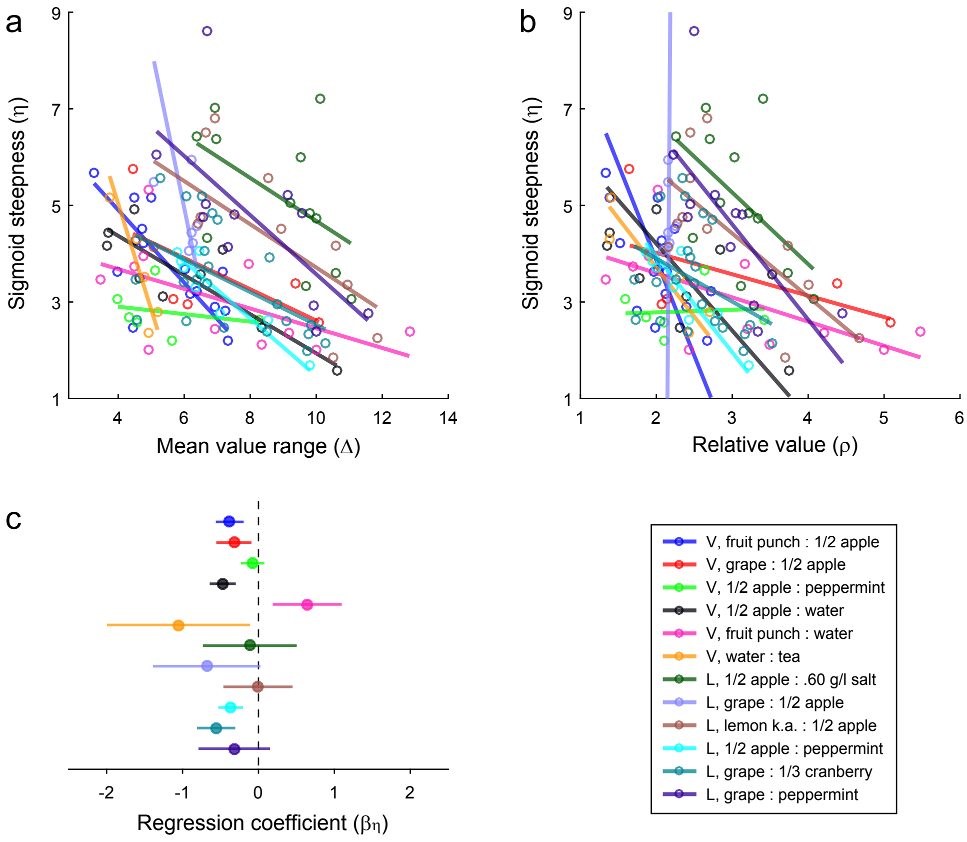

Supplementary Figure 6. Relation between sigmoid steepness (η) and value range (Δ), controlling for fluctuations in relative value (ρ). Because η, ρ and Δ are inter-related (see Methods), the relation between η and Δ (Fig.6) might simply reflect fluctuations in ρ. To address this issue, we regressed η on ρ and then on Δ in a stepwise way. The coefficient obtained from the second regression (β_η) quantified the correlation between η and Δ not explained by fluctuations of ρ. **a.** Relation between sigmoid steepness (η) and value range (Δ). This panel recapitulates Supplementary Figure 5. Different colors indicate different juice pairs (see legend). **b.** Relation between sigmoid steepness (η) and relative value (ρ). **c.** Results of stepwise regression. For most sets, we found $\beta_\eta < 0$, indicating that the residual η after regressing on ρ was still negatively correlated with Δ. Error bars indicate S.E.M. Considering the 12 sets, the distribution of β_η was significantly displaced from zero (mean(β_η) = -0.31, p = 0.01, one-tailed t test). In other words, the negative correlation between η and Δ was above and beyond the correlation explained by fluctuations of ρ. Color conventions in (b) and (c) are as in (a).

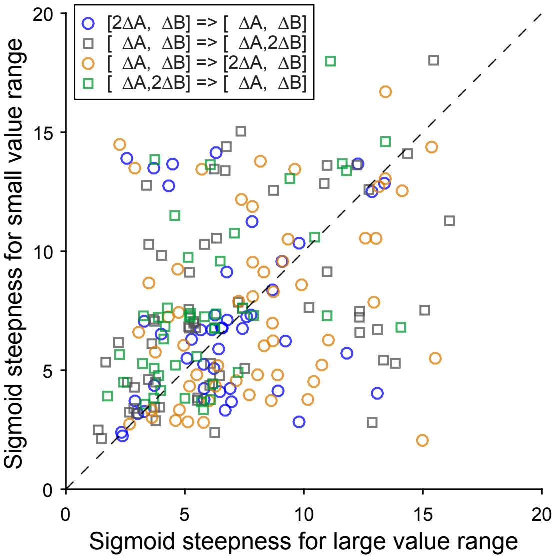

Supplementary Figure 7. Measures of sigmoid steepness in Exp.2. For each session, the two axes represent the steepness of the sigmoid measured with a large value range (x-axis) and that measured with a small value range (y-axis). Each data point represents one session. Different symbols represents the block order (see legend) and data from the two monkeys are pooled. Data points are broadly scattered, but overall they tend to lie above the identity line ($p = 0.04$, sign test). In other words, the sigmoid was steeper when value ranges were smaller. This effect was statistically significant when the analysis was restricted to sessions in which we varied the range of juice B ($p = 1.5$ 10-4, sign test), but not when the analysis was restricted to sessions in which we varies the range of juice A ($p = 0.37$, sign test). Sessions that presented perfect separation (saturated choice patterns) were excluded from this analysis.

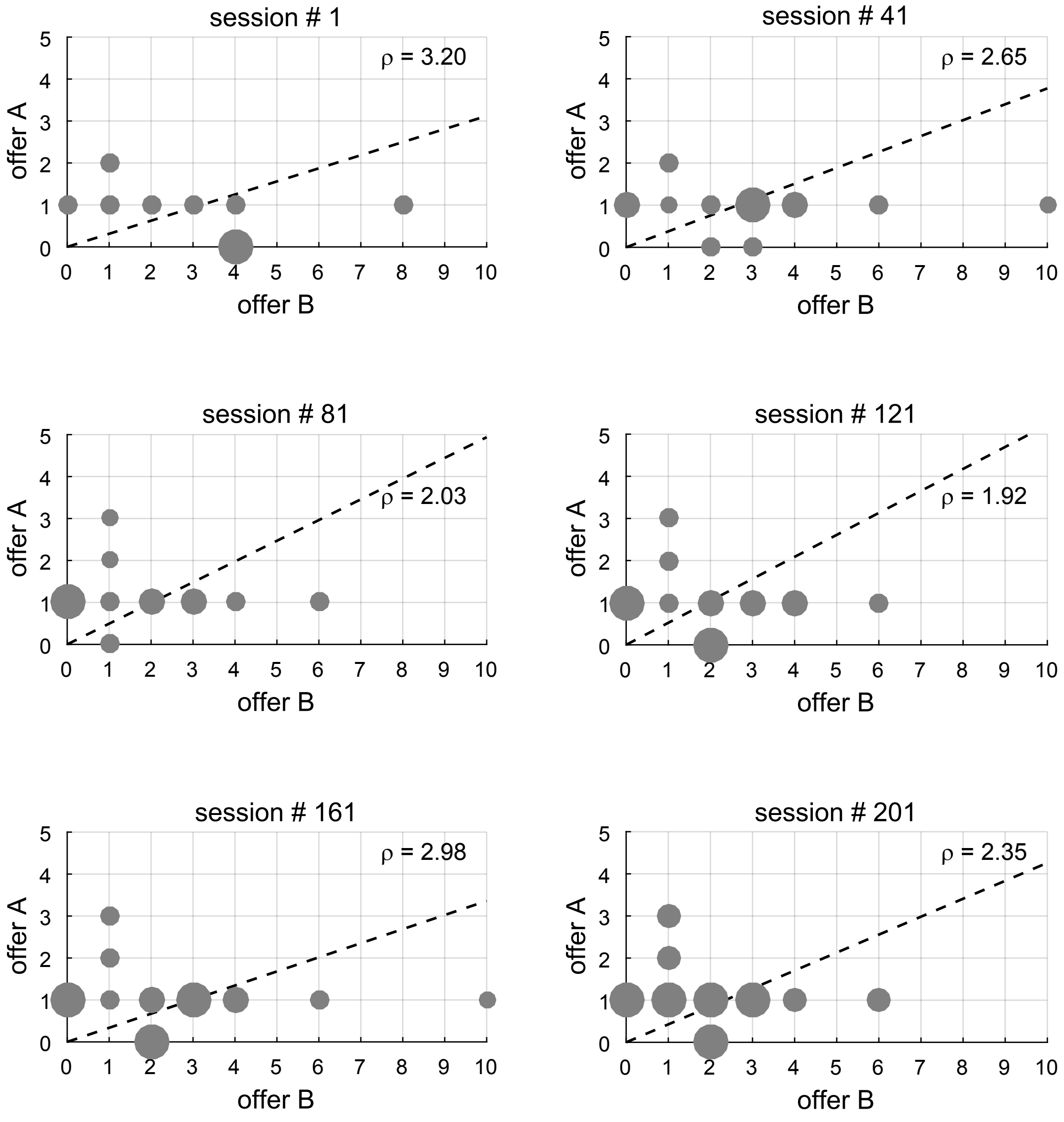

Supplementary Figure 8. Joint distributions of offers in Exp.1. The six panels illustrate the joint distribution of offers for six representative sessions (out of 208). For each session, gray dots represent offer types presented in the session and the dotted line represents the indifference line (ρ calculated with the sigmoid fit). The radius of each dot is proportional to #trials/max(#trials), where #trials is the number of trials for the corresponding offer type and max(#trials) is the maximum of #trials across offer types. For the six sessions, total number of trials was equal to 1,429 (session 1), 441 (session 41), 347 (session 81), 400 (session 121), 362 (session 161), and 228 (session 201). Note that the range of offer values and the relative multiplicity of trials for different offer types varied from session to session. Each session included forced choices for either juice. In the other trials, one of the two juices was always offered in quantity 1.

Variable X	Variable Y	R^2X	R^2Y	pval
offer A|B linear	*offer A|B* $ntrial_{CDF}$	88.8	27.4	$<10^{-8}$
offer A|B linear	*offer A|B* $ntrial_{CDF}$ *VE*	86.1	27.8	$<10^{-8}$
offer A|B linear	*offer A|B ORF*	69.6	29.2	$<10^{-5}$
offer A|B linear	*offer A|B mean(ORF)*	48.0	27.5	0.009

Supplementary Table 1. Variable selection, post-hoc analysis. The variable selection analyses were conducted twice. This table refers to the analysis that did not include variables based on $ORF_{uniform}$ (see Methods). In this case, both stepwise and best-subset methods selected variables *offer A linear*, *offer B linear*, *chosen value* and *chosen juice*. The table indicates the results of the post-hoc analysis based on collapsed variables (see Methods). To test whether the explanatory power of *offer A|B linear* (column X) was statistically higher than that of other variables examined in the analysis (column Y), we calculated the marginal explanatory power of each variable. For example, considering variables X and Y, the marginal explanatory power of X (column R^2X) was defined as the total R^2 explained by X but not by Y. The two variables were then compared with a binomial test. The results (p value in the last) indicate that the explanatory power of linear offer value variables was significantly higher than that of any competing variable.

Variable X	Variable Y	R^2X	R^2Y	pval
offer A|B $ORF_{uniform}$	*offer A|B linear*	37.7	32.5	0.270
offer A|B $ORF_{uniform}$	*offer A|B* $ntrial_{CDF}$	114.3	47.8	$<10^{-7}$
offer A|B $ORF_{uniform}$	*offer A|B* $ntrial_{CDF}$ *VE*	111.4	48.0	$<10^{-7}$
offer A|B $ORF_{uniform}$	*offer A|B ORF*	93.2	47.7	$<10^{-4}$
offer A|B $ORF_{uniform}$	*offer A|B mean(ORF)*	78.0	52.5	0.013

Supplementary Table 2. Variable selection, post-hoc analysis. This table refers to the analysis that included all the variables defined in this study, including those based on $ORF_{uniform}$. In this case, both stepwise and best-subset methods selected variables *offer A* $ORF_{uniform}$, *offer B* $ORF_{uniform}$, *chosen value* and *chosen juice* (see Fig.9). Same format as Supplementary Table 1. Note that all comparisons are statistically significant except that between *offer A|B* $ORF_{uniform}$ and *offer A|B linear*.

Optimal Coding and Neuronal Adaptation
in Economic Decisions

SUPPLEMENTARY NOTE

ALDO RUSTICHINI, KATHERINE E. CONEN, XINYING CAI,
AND CAMILLO PADOA-SCHIOPPA

Abstract. We formulate the general problem of optimal coding for a linear model for the choice with two pools of neurons. Optimal coding is defined as the problem of choosing the optimal response function for a given environment, under physiological constraints (such as a maximum firing rate), if one wants to maximize the expected value of the goods selected.

We consider first the case of a linear response function, and analyze the adjustment of the slope of the response to the distribution of offers in a session. Our aim is to solve two problems at the same time: explain why we observe adaptive coding (AC), and why AC does not induce a choice bias (that is, a dependence of choice on the environment: this is defined precisely in section 4.1).

We find:

(1) The optimal response requires adaptive coding (proposition 4.1 and theorem 5.2).
(2) If the firing rate and inputs have no upper limit, then the choice does not exhibit bias depending on variation of the range of the offers in different experimental sessions. There is no bias, but AC improves the response (proposition 4.1).
(3) If the firing rate and inputs have upper limits, then as the range of either good increases the stochastic choice becomes noisier (equation 26 in theorem 5.2).
(4) In both cases (upper bound or not) the consistency of choice is not a constraint imposed on the process, but an outcome of the optimization (proposition 4.1 and theorem 5.2).

In the second part we consider the general problem of characterizing the best response function in the set of all functions, not necessarily linear.

1. Choice environment

Two goods, A and B are offered during an experimental sessions. We use the index $g \in \{A, B\}$. In the main text we conducted the analysis in physical goods space: thus, a quantity q_A of good A and q_B of good B are offered, expressed in common physical units (say, ml), and $q_A \sim q_B$ (that is, the animal is indifferent between q_A and q_B) if and only if $\rho q_A = q_B$. This setup is closer to the nature of the neural coding (see Padoa-Schioppa (2009), in particular figure 4 of that paper), but is less natural for the analysis. So

Date: September 3, 2017.

Key words and phrases. Adaptive Coding, Economic Choice, Neuro-computational Theory.

in this supplementary material we work in value space, describing the two offers x for good A and y for good B in terms of a common value; so in this case $x \sim y$ if an only if $x = y$; so that we can take x to be ρq_A, and y to be q_B. The joint distribution of offers in a session is described by a $\pi \in \Delta(X \times Y)$; π_g is the marginal over quantities of good g.

2. Linear Model: Neuronal coding within sessions

We first describe the response in a trial of a given experimental session. There are two pools of neurons, one for each good g. Each set has the same number n of neurons; we will take the limit as $n \to \infty$ later. We consider the output of the two pools as providing input current to a post synaptic sets of neurons; so we will consider both the firing rate and the input to the downstream neuron for every spike; the latter is regulated by synaptic efficacy. We assume that all neurons in the pool fire at the same Poisson rate:

Assumption 2.1. *The spike process $r_{g,i}, g \in \{A, B\}$ for each neuron $i = 1, \ldots, n$ is Poisson with rate ν_g.*

We consider the total firing occurring in a fixed time interval Δt of approximate length 500 ms. There is no leak, thus all the spikes within this interval are interchangeable. We write the input corresponding to each spike of neurons of good g as J_g; this is the amount of positive charge entering the membrane of the post-synaptic neuron due to one spike. Since Δt does not play a role in our analysis, we choose it as time unit: $\Delta t = 1$. Now the firing rate refers to this time unit, and $r_{g,i}$ indicates the number of spikes per unit time. We define D^n as the random variable obtained as a weighted sum of the contributions of the two pools:

$$X_g^n \equiv \sum_{i=1}^{n} w_i^n r_{g,i} J_g \tag{1}$$

where w^n is a vector of n-positive weights, adding up to 1; and

$$D^n \equiv X_A^n - X_B^n \tag{2}$$

By the assumption 2.1

$$E(r_{g,i}J_g) = \nu_g J_g,\ Var(r_{g,i}J_g) = \nu_g J_g^2 \equiv \sigma_{r_g}^2 J_g^2 \tag{3}$$

We focus here on the simple case where

$$\forall i, w_i^n \equiv \frac{1}{n}. \tag{4}$$

We want to take limits as $n \to \infty$, defining in the limit a random variable D. Assuming stationarity (in the spatial dimension index) then by Birkhoff ergodic theorem, a limit random variables D exists, which is the expectation of the representative random variable $r_{A,1}J_A - r_{B,1}J_B$ with respect to the invariant σ-algebra.

The expectation of this limit variable is:

$$E(D) = \nu_A J_A - \nu_B J_B \tag{5}$$

The variance of D^n is

$$Var(D^n) = \frac{1}{n}(J_A^2 Var(r_{A,1}) + J_B^2 Var(r_{B,1})) + \tag{6}$$

$$(1 - \frac{1}{n}) \left(J_A^2 Cov(r_{A,1} r_{A,2}) + J_B^2 Cov(r_{B,1} r_{B,2})\right) - 2J_A J_B Cov(r_{A,1} r_{B,1})$$

We assume (on the basis of experimental evidence: see figure S4 of the text) that the correlation among neurons is independent of the firing rate ν and the vector of inputs J:

Assumption 2.2. *$Corr(r_{g,1} r_{g,2}) = \chi_g, g = A, B$ and $Corr(r_{A,1} r_{B,1}) = \gamma$, all independent of the vectors rate ν and input J.*

Using 2.2 we get:

$$Var(D) = \chi_A \sigma_{r_A}^2 J_A^2 + \chi_B \sigma_{r_B}^2 J_B^2 - 2\gamma \sigma_{r_A} J_A \sigma_{r_B} J_B \tag{7}$$

Note that when $\chi_A = \chi_B \equiv \chi$ and $\sigma_{r_A}^2 = \sigma_{r_B}^2 \equiv \sigma^2$ and $J_A = J_B$, then $\chi \geq \gamma$, so correlation within pools is larger than the one across pools. In the analysis below we set $\gamma = 0$ for simplicity of notation; introducing it back is trivial.

We assume that good A is chosen if $D \geq 0$, and B otherwise. This *linear decision model* is a very reduced form of mutual and of pooled inhibition models.

3. Adaptive coding across sessions

We consider the problem of the adjustment of the neuronal response from one session to another. Neurons corresponding to a good respond in each trial to the quantity offered of the corresponding good; the function describing this response can vary across sessions, depending on π. We now consider different possible restrictions on this dependence.

3.0.1. *Flexibility of parameters.* First we assume that the input J does not depend on the quantities offered in the trial:

Assumption 3.1. *A choice of response in a session is a pair of functions assigning rate response and input to a distribution, $(\nu_g(\cdot|\pi), J_g(\pi))$, where $\nu_A(x|\pi)$ is the rate associated with the quantity x of A, and so on; $J_g(\pi)$ is a real number.*

We justify this restriction arguing that the synaptic efficacy J can be modified by learning during the session in a way that depends on π (for example through Hebbian learning) but cannot adjust quickly enough to trial by trial variations in the quantities offered.

A second restriction is that:

Assumption 3.2. *For both goods g, the rate function ν_g and input J_g only depend on π_g (and not on the marginal on the other good).*

If this assumption holds, at the beginning of the session the pool of g-neurons is "informed" of π_g, can adjust to it, but its response cannot depend on $\pi_{g'}$, $g' \neq g$.

We will assume both assumption 3.1 and assumption 3.2 in the following.

4. Maximization problem

Fix π and a choice $(\nu, J) = (\nu_g, J_g)_{g\in\{A,B\}}$. For any given pair of quantities (x, y) we have a probability distribution on the real line, $Q(\cdot|(x, y), \nu, J)$. The problem we consider is the maximization of the expected payoff:

$$\int_{X\times Y} (Q(R^+|((x, y), \nu, J))x + Q(R^-|((x, y), \nu, J))y)d\pi(x, y) \tag{8}$$

where R^+ and R^- are the sets of positive and strictly negative real numbers, and π is the joint distribution defined at the end of section 1. So our problem is:

$$\max_{(\nu,J)} \int_{X\times Y} (Q(R^+|((x, y), \nu, J))x + Q(R^-|((x, y), \nu, J))y)d\pi(x, y) \tag{9}$$

where (ν, J) satisfies the appropriate restrictions, depending on assumptions 3.1 and 3.2. After a simple rearrangement we have that (ν, J) is a solution of 9 if and only if it is a solution of 10:

$$\max_{(\nu,J)} \int_{X\times Y} (Q(R^+|((x, y), \nu, J))(x - y)d\pi(x, y) \tag{10}$$

From our analysis in section 2, using the formula for expectation and variance of D we obtain the crucial information that the mean and variance of the limit random variable scale as in equations 5 and 7. We have then:

$$Q(R^+|((x, y), \nu, J)) = \tag{11}$$

$$Pr\left(Z \geq -\frac{\nu_A(x|\pi)J_A(\pi) - \nu_B(y|\pi)J_B(\pi)}{(\chi_A\nu_A(x|\pi)J_A^2(\pi) + \chi_B\nu_B(x|\pi)J_B^2(\pi))^{1/2}}\right)$$

where Z is a standardized random variable with mean 0 and variance 1. We assume for computational convenience, particularly in the numerical simulations, that the random variables D and Z are normal. A more detailed description on D (beyond the information on mean and variance, which are the key elements in our analysis below) requires a full model of the the network, that will give account of the long distance correlation among neurons. This full model, allowing for long distance positive correlation (and not only asynchronous state, as in Renart et al. (2010) or Rosenbaum et al. (2017)), is topic of current research.

Noting that the lower bound in the equation above is homogeneous of degree zero in J, we reformulate the problem as maximization in ν and $R \equiv \frac{J_B}{J_A}$ where the probability in equation 11 has the form:

$$Pr\left(Z \geq -\frac{\nu_A(x|\pi) - \nu_B(y|\pi)R(\pi)}{(\chi_A \nu_A(x|\pi) + \chi_B \nu_B(x|\pi) R^2(\pi))^{1/2}}\right). \tag{12}$$

4.1. **Choice bias.** Our analysis will provide a model for the probability that the animal chooses good A if the pair offered is (x, y) and the probability on goods in the session is π, call it $P(Ch = A|x, y, \pi)$. We say that there is no choice bias if two conditions are satisfied.

The first (equation 13) is that this probability only depends on the pair (x, y) and not on π (as long as, of course, the pair (x, y) is in the support of the distribution).

$$\forall \pi, \pi', x, y : P(Ch = A|x, y, \pi) = P(Ch = A|x, y, \pi') \tag{13}$$

The condition 13 is not restrictive enough. For example, choosing A in all trials of all sessions satisfies 13, so we add:

$$\forall \pi : P(Ch = A|x, y, \pi) \text{ is increasing in } x, \text{ decreasing in } y \text{ and equal to } 1/2 \text{ if } x = y. \tag{14}$$

4.2. **Functional restrictions.** So far we have constrained the response functions ν only through their dependence on π. We now consider the additional restriction of linearity:

$$\nu_A(x|\pi) = s_A(\pi)x, \nu_B(y|\pi) = s_B(\pi)y \tag{15}$$

where $s_g(\pi) \in R^+$. The constraint 15 is very strong; we discuss its weakening in section 6 below.

We define the largest value in the support of the distribution of a good: $M_g(\pi) \equiv \max\{z \in \mathit{support}(\pi_g)\}$; we may drop the π in the notation if this dependence is clear. Consistently with experimental evidence, we assume that $\chi_A = \chi_B = \chi$. With the linearity assumption 15 we say that there is full linear adaptive coding if there is a single $s > 0$ such that $s_g = \frac{s}{M_g}$ for all g. In this case if $M_A = M_B$ then $\nu_A(x|\pi) = sx, \nu_B(y|\pi) = sy$, and the formula 11 above becomes:

$$Q(R^+|((x, y), s, J)) = Pr(Ch = A|x, s, J) = \tag{16}$$
$$Pr\left(Z \geq -\frac{J_A x s_A - J_B y s_B}{\sqrt{\chi(J_A^2 x s_A + J_B^2 y s_B)}} | Z \sim N(0, 1)\right).$$

which is equation (2) in the main text. Recall that x and y are value units, so firing rate and synaptic efficacy for each good change accordingly. Setting the correlation χ notionally equal to 0 clarifies the scaling in both cases. When using physical units, we want the effective firing rate (firing rate t_g times synaptic efficacy (call it K_g) to be equal when the animal is indifferent between the two offers, that is $t_A K_A q_A = t_B K_B q_B$ if and only if

$\rho q_A = q_B$. This holds if and only if $\frac{t_A}{\rho} K_A = t_B K_B$. In value units we want the effective firing rate to be equal ($s_A J_A x = s_B J_B y$) if and only if $x \sim y$, that is $x = y$, and this occurs if and only if $s_A J_A = s_B J_B$. Thus, the pair (x, y) here corresponds to $(\rho q_A, q_B)$ in the main text; the pair (J_A, J_B) here to (K_A, K_B) in the main text, the pair (s_A, s_B) to the pair $\left(\frac{t_A}{\rho}, t_B\right)$, and finally the pair (M_A, M_B) to the pair $(\rho Q_A, Q_B)$.

Proposition 4.1. *If the maximization problem satisfies equation 15 and there is no constraint on the firing rate, then with full linear adaptive coding:*

$$s_g \equiv \frac{s}{M_g}, J_g = M_g \tag{17}$$

(1) *As $s \to \infty$, the expected payoff tends to the maximum possible value given by:*

$$\int_{X \times Y} \max\{x, y\} d\pi(x, y),$$

so there is no choice bias (that is, both equations 13 and 14 hold).

(2) *If $J_A = J_B$ the expected payoff in strictly increasing in $\sqrt{s}$.*

Proof. Under our assumption and functional form of the response the quantities in 11 are equal to

$$Pr(Ch = A|x, y, \pi) = Pr\left(Z \geq -\left(\frac{s}{\chi(xM_A + yM_B)}\right)^{1/2}(x - y)\right) \tag{18}$$

so as $s \to \infty$ the probability of choosing $\max\{x, y\}$ tends to 1 for all (x, y), independently of π.

The statement (2) follows differentiating the expected payoff with respect to $\sqrt{s}$; the derivative is the integral of a positive function. □

The restriction $J_A = J_B$ is needed here because if $\frac{J_B}{J_A} > 1$, say, and π is concentrated on the sector of (x, y) such that $\frac{J_B}{J_A} y > x > y$ then the derivative with respect to $\sqrt{s}$ may be negative (as intuitively clear: increasing $\sqrt{s}$ reduces the probability of the A choice (because $J_A x - J_B y < 0$), when $x > y$.)

4.3. **Re-scaling of χ.** We clarify now the details linking the current analysis to the data, to justify setting $\chi = \xi/4$ as in the main text, immediately below equation (2). There are several different re-scalings that we use in data analysis; they can all be conveniently represented by a multiplication of the correlation coefficient χ by a constant c. There are two re-scalings in particular that need attention.

The first re-scaling is produced by the fact that we use in the analysis the time unit of the decision time (called Δt in section 2). The crucial point here is that the variable D^n defined in 2 considers the spikes in the time interval Δt, and **not** in 1 second units. This is convenient because we can ignore this Δt. Suppose this decision time is 0.5 seconds. Then if we use

the firing rate in seconds (Hz), we have to adjust to compensate for the different time units. For example if the firing rate in decision time units is 8, then the one in Hz is 16. If we replace the firing rate in Δt units with the one in units of seconds, we have a different formula, where the correlation coefficient is multiplied by $c = 2$, or in general by a factor $\frac{1}{\Delta t}$.

The second re-scaling is used in the numerical analysis of the linear response model. Here we take $\nu_A = s_A x$, for example. We constrain the slope s_A to reflect the constraint that the firing rate cannot be larger than a maximum $\overline{\nu}$ for any quantity x, so we require

$$s_A M_A \leq \overline{\nu}.$$

In our numerical simulations reported below we formulate the constraint for convenience not $s_A \leq \frac{\overline{\nu}}{M_A}$ but as $s_A \leq 1$. Again we can do this but we need to re-scale the firing rate, because we are implicitly defining the new slope as $s_A \frac{M_A}{\overline{\nu}}$, and we need to introduce an adjustment by introducing a scaling factor c. In the numerical analysis the M_A is given by the maximum number of units of the good presented; the maximum rate $\overline{\nu}$ has to be estimated by looking at the difference between the largest firing rate we observe and the baseline firing rate, which in Hz is approximately 8 (see Padoa-Schioppa (2013)). The multiplication by 2 and the division by 8 in the two re-scalings explain the correction $\chi = \xi/4$.

5. Constraints on the responses

Of course the limit in $s \to \infty$ is biologically meaningless. In this section we reconsider the maximization problem presented in section 4, but with the assumption that the responses are linear, and introducing explicitly the constraints on the responses. So we solve:

$$\max_{(s_A, J_A, s_B, J_B)} \int_{X \times Y} \left(\int_{-L}^{\infty} \phi(z) dz \right) (x - y) d\pi(x, y) \tag{19}$$

(where

$$L = \frac{s_A J_A x - s_B J_B y}{(\chi(s_A J_A^2 x + s_B J_B^2 y))^{1/2}} \tag{20}$$

ϕ is the density of the standard normal), subject to:

$$\forall g,\ 0 \leq s_g \leq \frac{\overline{\nu}}{M_g}. \tag{21}$$

The constraint 21 insures that the firing rate at all quantities offered of the good is smaller than a maximum firing rate $\overline{\nu}$. The following also may seem natural:

$$\forall g,\ 0 \leq J_g \leq \overline{J} \tag{22}$$

but as we noted the value of L in equation 20 is homogeneous of degree zero in the J inputs; so re-scaling them by a common positive factor does not affect choice. The lower integration L can be rewritten as:

$$L = \frac{s_A x - s_B R y}{(\chi(s_A x + s_B R^2 y))^{1/2}} \tag{23}$$

where $R \equiv \frac{J_B}{J_A}$, constrained only by:

$$R \geq 0. \tag{24}$$

The original problem is equivalent to maximize 19, with L as in 23, subject to the two constraints 21 and 24. Once an optimal triple (s_A, s_B, R) has been determined, pairs of 22-feasible inputs (J_A, J_B) can be found, the optimal J's are indeterminate.

5.1. **Symmetric distribution.** We begin with a simple case, the symmetric uniform distribution on offers.

Theorem 5.1. *Let $M_A = M_B \equiv M$ and π be the product of the uniform distribution on $[0, M]^2$. The optimal linear response is:*

$$(s_A, s_B, R) = \left(\frac{\overline{\nu}}{M}, \frac{\overline{\nu}}{M}, 1 \right) \tag{25}$$

The probability of choice of good A with offers (x, y) at the optimal solution is

$$Pr(Ch = A | x, y, \pi) = Pr\left(Z \geq - \left(\frac{\overline{\nu}}{\chi M (x + y)} \right)^{1/2} (x - y) \right) \tag{26}$$

Proof. Write the Lagrangean and solve. □

At $s_A = s_B \equiv s$ the values of the net payoff (maximand in 19) for different values of s and R is displayed in Supplementary Note Figure 1. The figure confirms that (for $s = 1$) the local maximum at $R = 1$ is a global maximum.

5.2. **Non-symmetric, uniform distribution.** We consider here the more general case of a uniform distribution but on two possibly different ranges. To understand the shape of the optimal solution in this case it is first useful to consider the basic trade-offs in the problem. To fix ideas we consider the case in which $M_B > M_A$.

If we consider the lower integration term L in equation 20, we note that the mean scales in the firing rate slope s_A and s_B linearly, whereas the standard deviation (the denominator in L) scales as the square root; hence we may want to make the slopes as large as possible. The slopes are bounded by a maximum, and the maximum slope is decreasing in the range (equal to $\overline{\nu}/M_g$); hence once we set them to a maximum we are introducing a potential bias in the choice, making the probability of choosing the good B smaller than it should be. The J inputs can correct this; as we noted their scale is irrelevant (both mean and SD scale linearly in the inputs) so we can correct

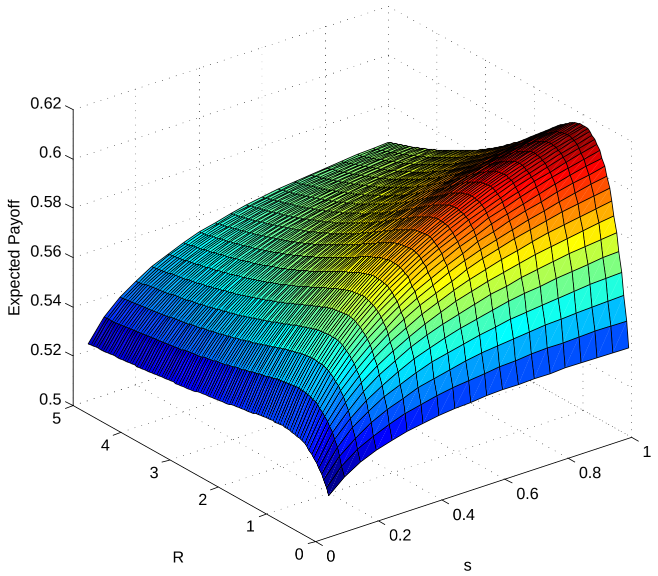

Supplementary Note Figure 1. **Surface of the expected payoff as function of (s, R).** Value of s satisfy the constraint in (21); R is unconstrained above but truncated at $R \leq 7$. Here we set $s_A = s_B \equiv s$, $M_A = M_B = 1, \overline{\nu} = 1, \chi = 0.02$. The optimal solution is at $(s, R) = (1, 1)$.

entirely the bias, setting $R = M_B/M_A$. This describes an "almost optimal" solution, depending on the value of χ. However, this is not necessarily the optimal solution, and this depends on the value of the correlation χ.

To understand why the proposed solution is not precisely optimal, and the role of the correlation χ, one can compare this solution to the policy of "always choosing B". This policy chooses the wrong option (hence possibly inducing a loss compared to the "no-bias" policy) only when $x > y$, which, as M_B becomes large compared to M_A, is unlikely; on the rest of the offers region it chooses the right option for sure (hence a gain, compared to the "no-bias" policy). When the noise is large, the gain overcomes the loss. The true optimal solution adopts some of the "always choosing B", introducing a small bias in favor of the option B, by making $R > \frac{M_B}{M_A}$. This is better than the no bias solution, because in this way we balance the small cost of the bias in the triangular region between $y = x$ and $y = R\frac{M_B}{M_A}x$, and the gain of increasing the probability of the B choice over the *entire region* $y > x$.

Theorem 5.2. *Let π be the product of the uniform distribution on $[0, M_A] \times [0, M_B]$. The optimal linear response is:*

$$(s_A, s_B) = \left(\frac{\overline{\nu}}{M_A}, \frac{\overline{\nu}}{M_B} \right) \tag{27}$$

If we let $m \equiv \frac{M_B}{M_A}$, we note that the ratio $\frac{R}{m}$ has a natural interpretation of a bias. To see this, consider that a bias is absent only if the probability of choice of A is equal to $1/2$ when $x = y$; that is when the lower extreme of integration is $L = 0$; this in turn is true if and only if $x = \frac{R}{m} y$. So for the probability of choosing A over B to be exactly $1/2$ when $x = y$ it is necessary that $\frac{R}{m} = 1$.

In table 1 we report the bias in favor of the good B (a positive number indicates that the good B is more likely to be chosen when $x = y$) at the optimal values of R. The dependence of the optimal R on χ and M_B is illustrated in Supplementary Note Figure 2 for a range of χ closer to the empirically relevant value or larger. The optimal R is 1 at $M_B = M_A$, $R > M_B/M_A$ for $M_B > M_A$, and $R < M_B/M_A$ for $M_B < M_A$. As χ becomes larger, the optimal R deviates more from M_B/M_A.

Table 1. **Bias as function of ρ and M_B.** In the table $M_A = 1$, $\overline{s} = 1$. The entries denote the bias in favor of the B good, that is the value $\frac{R}{M_B} - 1$ (see the remarks after the statement of the theorem 5.2). The values of χ (ranging from 0.008 to 0.013) are indicated in the top row; the values of M_B (ranging from 0.50 to 1.50) on the left column.

	0.008	0.009	0.009	0.010	0.010	0.010	0.011	0.011	0.012	0.012	0.013
0.5	-0.04	-0.04	-0.04	-0.04	-0.04	-0.05	-0.05	-0.05	-0.05	-0.05	-0.06
0.6	-0.03	-0.03	-0.04	-0.04	-0.04	-0.04	-0.04	-0.04	-0.05	-0.05	-0.05
0.7	-0.03	-0.03	-0.03	-0.03	-0.04	-0.04	-0.04	-0.04	-0.04	-0.04	-0.04
0.8	-0.03	-0.03	-0.03	-0.03	-0.03	-0.03	-0.03	-0.03	-0.03	-0.04	-0.04
0.9	-0.02	-0.02	-0.02	-0.02	-0.02	-0.02	-0.02	-0.02	-0.02	-0.02	-0.02
1	0.00	0.00	0.00	0.00	0.00	0.00	0.00	0.00	0.00	0.00	0.00
1.1	0.02	0.02	0.02	0.02	0.02	0.02	0.02	0.02	0.02	0.02	0.02
1.2	0.02	0.02	0.03	0.03	0.03	0.03	0.03	0.03	0.03	0.03	0.03
1.3	0.03	0.03	0.03	0.03	0.03	0.03	0.04	0.04	0.04	0.04	0.04
1.4	0.03	0.03	0.03	0.03	0.04	0.04	0.04	0.04	0.04	0.04	0.05
1.5	0.03	0.03	0.03	0.04	0.04	0.04	0.04	0.04	0.05	0.05	0.05

5.3. **Stochastic Choice.** As ranges change, the slope of the stochastic choice function changes. In the symmetric case ($M_A = M_B$), from equation 26, we have that (here $\pi = 3.1415\ldots$, not the distribution over goods):

$$\text{slope of the sigmoid at } x = y \text{ is } \left(\frac{\overline{\nu}}{2\pi\chi x(M_A + M_B)} \right)^{1/2}. \tag{28}$$

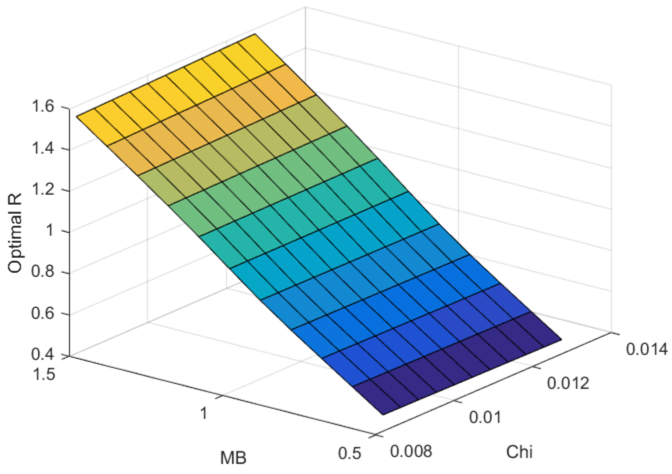

SUPPLEMENTARY NOTE FIGURE 2. **Surface of the optimal R as function of M_B and χ.** Here $M_A = 1, \overline{\nu} = 1$.

We write explicitly M_A and M_B for ease of comparison with the non symmetric case.

In the non symmetric case we can compute the slope on the line in the $x - y$ space where the probability of choice is equal to 1/2, that is on the line where

$$J_A s_A x - J_B s_B y = 0. \tag{29}$$

because this is where we are going to compute the slope in the empirical estimates. Due to the bias which is evident in Supplementary Note Figure 2, this is not the line $y = x$; the function giving the value y indifferent to x as a function of x is flatter when $M_B > M_A$. In this case:

$$\text{slope at } (x, y) \text{ where (29) holds is } \left(\frac{\overline{\nu}}{2\pi\chi x(1+R)M_A}\right)^{1/2}. \tag{30}$$

Note that by our results on the optimal R (see remarks after theorem (5.2), the slope is less than $\left(\frac{\overline{\nu}}{2\pi\chi x(M_A+M_B)}\right)^{1/2}$ when $M_B > M_A$. The extent of the flattening of the slope is provided by the equation 30, considering the values of R given in Supplementary Note Figure 2.

This estimate of the slope provides an easy test of the model through the dependence on the ranges of observed choices.

5.4. **Payoff and Range.** We now examine how the expected payoff changes when the ranges of the two goods change.

5.4.1. *Re-scaling the range.* In the symmetric case, consider first the effect on the expected payoff of increasing the range, while keeping the symmetry property. The expected payoff is homogeneous of degree one in the range: if we write the expected payoff at the optimal solution in the range $[0, TM]^2$ as $Pay(TM)$ then $Pay(TM) = TPay(M)$. Thus, doubling the range doubles the payoff. This is clear from a change of variable, considering the formula for the lower term of integration L in equation (20). Consequently also the ratio between the expected payoff at the optimal solution and the maximum payoff does not change as the range is re-scaled. This is clear by the fact that the maximum payoff too is homogeneous of degree 1 in the scaling factor T. Note that the slope of the response decreases when the range increases.

5.4.2. *Conditional Payoff.* Instead, the conditional payoff decreases as the slope decreases. To be precise, let $M \equiv M_A = M_B$, and re-scale M by a factor $T \geq 1$. For any T we can compute the conditional expected payoff from the choices made when x and y are in the range $[0, M]^2$, but with the slope optimal for the range $[0, TM]^2$. The conditional expected payoff is strictly decreasing in T for $T > 1$; as one can check the derivative with respect to T is given by

$$-\frac{1}{2T^{3/2}M^2}\int_{[0,M]^2}\phi(-L)\frac{(x-y)^2}{(\chi(x+y))^{1/2}}dxdy.$$

hence strictly negative. The payoff as $T \to +\infty$ tends of course to the expected payoff from the random choice with equal probability on each option.

5.4.3. *Comparison with the maximum payoff.* Supplementary Note Figure 3 displays the ratio of the expected payoff at the optimal choice of slopes and R ratio over the expectation of the maximum payoff (obtained by choosing the maximum of x and y at every point). Clearly the worst situation for the optimal choice is the symmetric case, where the payoff is smallest for any fixed value of χ. Note that for realistic values of χ close to the empirically relevant value the deviations are very small. This is natural, as the payoff from the constant policy "always choose the option with the largest range" tends to the maximum payoff as the ratio between the two ranges increases to infinity, and the optimal policy becomes close to the constant policy as the range of one of the goods increases. As natural, the ratio decreases with the correlation χ.

6. Non-linear response functions

If we take the point of view of section 3 that the slope is chosen to maximize expected payoff it is natural to enquire what the best response would be if the variable is the function instead of the slope, with no other constraint than the bound of the firing rate.

A similar optimization criterion underlies the alternative Barlow-Simon (BS) hypothesis, that the response function is the cumulative distribution function of probability distribution of the stimulus in the environment. It is

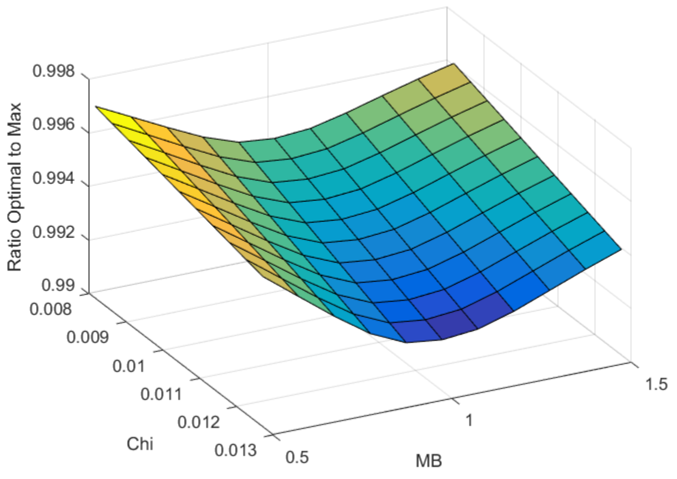

Supplementary Note Figure 3. **Ratio of the expected payoff at the optimal slope and optimal R over the maximum payoff.** Here $M_A = 1, \overline{\nu} = 1$.

well known that this response function is the optimal solution of a clearly defined information theoretic problem, namely maximizing the entropy of the distribution of the output signal over the response functions. The statement follows from two observations. First, the unconstrained maximum entropy over the output space (that is, the maximum entropy over *all* distributions) is achieved by the uniform distribution. Second, the uniform distribution over the output space can be achieved precisely, by setting the response function to be the *cdf* of the signal (when the density is no-where zero). If the objective function is not the entropy but the expected payoff the answer may well be different, and for a good reason, as we will see (section 6.1). The problem we consider is the one presented in section 4, with no restriction on the ν_g response functions.

6.1. **Intuitive Properties of the Optimal Solution.** To understand the fundamental feature of the optimal solution we can consider a simple example. The good A is offered in a fixed quantity of 1/2. The good B has offers in quantity $y \in \{0, a, 1\}$ with probabilities p_1, p_2 and p_3. To recover the case of a no-where zero density we can if necessary combine this with a uniform distribution over the unit interval. A function f from the space Y of quantities of B and the unit interval in the signal space Z can be chosen. A signal equal to $f(y) + \epsilon$ is observed, where $\epsilon \sim N(0, \sigma_\epsilon^2)$. The optimal choice policy is clearly described by a threshold value z_{th} and the choice of B when the observed signal is larger than the threshold. The optimal f is extreme valued. First, it clearly sets $f(0) = 0$, $f(1) = 1$. If we think of σ_ϵ^2 as small, it is clear that the optimal $f(a)$ is 0 when $a < 1/2$ (so the probability of the

signal crossing the threshold is minimal) and symmetrically 1 when $a > 1/2$. Now, adding several intermediate points a_i between 0 and 1 will not change the general S-shaped feature: all values below $1/2$ will be mapped to 0, and those above to 1; in general, low values and high values are lumped into similar signals, and the optimal function f is S-shaped. The BS solution is very different: the function f has three sharp increases at each of the three values of y, independently of the relative position of a and $1/2$.

The example illustrates the fundamental difference between the BS problem and the problem stated in section 4. Even if one does not accept the criterion we suggested in section 4, the BS solution has some revealing and counterintuitive features. First, the BS solution is only trying to maximize the sharpness of the local resolution, and is not interested in the global property of the distribution. This is obvious if one considers that the BS solution consists in setting the derivative of the response function equal to the density, a completely local definition (the value of the density at one point defines completely the derivative of the firing rate function at that point). Second, the BS is defined *only* by the properties of the environment and is not interested in the properties of the system that is making the choice, as none of the properties of this system enters into the solution. For instance it does not consider the signal to noise ratio, or the correlation coefficient χ which is crucial instead to define the optimal solution to the problem stated in section 4. These are obvious consequences of the fact that there is no *choice* problem this policy is solving.

In summary, it should be clear that the BS solution is irrelevant in the context of economic choice problems.

6.2. **Results.** Two main ideas describe the optimal response. One can isolate the first by considering first the symmetric case, where the distribution on the product of space of the two goods is the product of the marginals, and then proceeding to consider the non symmetric case in which the two marginals are not the same.

6.2.1. *Symmetric distributions.* By symmetry, the optimal solution will have $\nu_A = \nu_B \equiv \nu$ and $R = 1$. The optimal ν is displayed in Supplementary Note Figure 4, for different values of the correlation coefficient. The best response function has several main properties.

It is non-linear, S-shaped, with two almost flat regions at the low and high level of offers, and an intermediate curve approximately convex. The function depends on the correlation χ, becoming steeper in the intermediate ranges as χ increases. For low values of χ the flat regions are absent, and the function is monotonically strictly increasing, convex. Supplementary Note Figure 4 illustrates these two properties.

6.2.2. *Non-Symmetric distributions.* When the distribution on the product space is not the product of two identical distributions the value of R may be different from 1. As we have seen in the case of the linear response function,

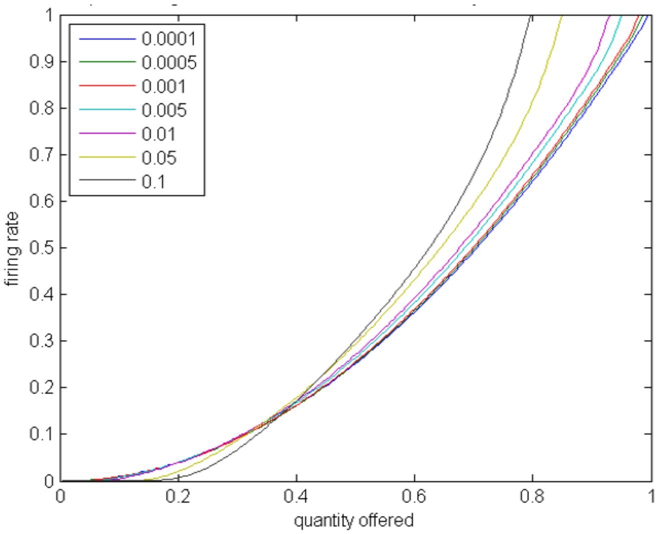

Supplementary Note Figure 4. **Optimal response function and correlation coefficient.** Here $M_A = M_B = 1, \overline{\nu} = 1$; the values of the correlation coefficient χ are indicated in the window. Grid of 200 values in the offer space.

the difference $s_A J_A - s_B J_B$ is a measure of the bias, in favor of good A if the difference is positive.

For small values of χ and of the difference between the two distributions, the optimal solution is of course close to the solution of a symmetric problem. For larger values of both variables, R is substantially different from 1, but the optimal firing rates and optimal R are adjusted so that in the region of offers $[0, 1] \times [0, 1]$ the bias is minimal. Supplementary Note Figure 5 illustrates.

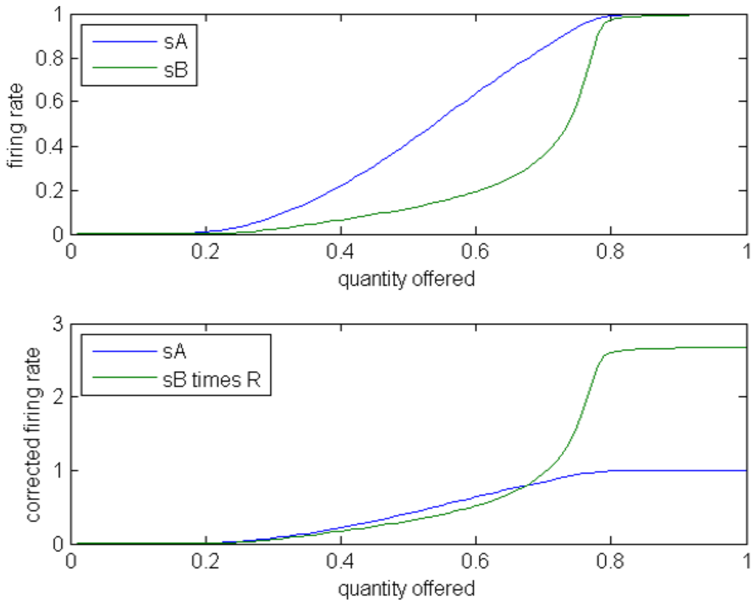

Supplementary Note Figure 5. **Best response functions, asymmetric case.** $M_A = 1, M_B = 1.5, \chi = 0.1$. Top panel: best responses; the range of values of the quantity offered is limited to the interval between 0 and 1 (the range for good A) to make the comparison between the two best responses easier. Bottom panel: the s_B function is multiplied by the optimal $R = 2.682$.

Supplementary References

Padoa-Schioppa, C. (2009): "Range-Adapting Representation of Economic Value in the Orbitofrontal Cortex," *Journal of Neuroscience*, 29, 14004–14014.

——— (2013): "Neuronal Origins of Choice Variability in Economic Decisions," *Neuron*, 80, 1322 – 1336.

Renart, A., J. de la Rocha, P. Bartho, L. Hollender, N. Parga, A. Reyes, and K. D. Harris (2010): "The Asynchronous State in Cortical Circuits," *Science*, 327, 587–590.

Rosenbaum, R., M. Smith, A. Kohn, J. Rubin, and B. Doiron (2017): "The spatial structure of correlated neuronal variability," *Nature Neuroscience*, 20, 107–114.
